# The development and validation of an index to predict 10-year mortality risk in a longitudinal cohort of older English adults

**DOI:** 10.1093/ageing/afw199

**Published:** 2016-10-28

**Authors:** Lindsay C. Kobayashi, Sarah E. Jackson, Sei J. Lee, Jane Wardle, Andrew Steptoe

**Affiliations:** 1 Center for Population and Development Studies, Harvard T.H. Chan School of Public Health, Harvard University, Cambridge, MA, USA; 2 Department of Epidemiology and Public Health, Institute of Epidemiology and Health Care, University College London, London, UK; 3 Division of Geriatrics, School of Medicine, University of California San Francisco, San Francisco, CA, USA

**Keywords:** older people, mortality risk, risk factors, mortality risk calculator, 10-year mortality risk

## Abstract

**Background:**

we aimed to develop and validate a population-representative 10-year mortality risk index for older adults in England.

**Methods:**

data were from 10,798 men and women aged 50 years and older in the population-based English Longitudinal Study of Ageing in 2002/03, randomly split into development (*n* = 5,377) and validation cohorts (*n* = 5,421). Participants were asked about their sociodemographics, health behaviours, comorbidities, and functional status in the home-based interviews. Variables that were independently associated with all-cause mortality through March 2013 in the development cohort were weighted relative to one another to develop risk point scores for the index that was calibrated in the validation cohort.

**Results:**

the validated 10-year mortality risk index assigns points for: increasing age (50–59 years: 0 points; 60–64: 1 point; 65–69: 3 points; 70–74: 5 points; 75–79: 7 points; 80–84: 9 points; ≥85: 12 points), male (2 points), no vigorous physical activity (1 point), smoking (2 points), having a diagnosis of cancer (1 point), chronic lung disease (2 points) or heart failure (4 points), and having difficulty preparing a hot meal (2 points), pushing or pulling large objects (1 point) or walking 100 yards (1 point). In the full study cohort, 10-year mortality rates increased from 1.7% (11/664) in those with 0 points to 95% (189/199) among those with ≥16 points.

**Conclusion:**

this highly predictive 10-item mortality risk index is valid in the English population aged 50 years and older. It uses simple information that is often available in research studies and patient reports, and does not require biomarker data to predict mortality.

## Introduction

As the age structures of global populations shift upwards, the ability to predict mortality risk for older adults becomes increasingly valuable in clinical, research and policy settings. Risk prediction scores are used extensively in the cardiovascular context, but the plethora of cardiovascular risk calculators remain invalidated and are restricted to a single clinical category [1, 2]. Mortality risk calculators have broader applicability, such as aiding older patients and their physicians in making decisions informed by precise estimations about remaining life expectancy [2–4]. In epidemiological research, mortality risk could be adjusted in observational and randomised studies, examined in relation to exposures or treatments, or compared across sociodemographic groups to assess social inequalities. For these uses to be achievable, an appropriate mortality risk index for older adults should include information that is readily accessible through physician–patient discussion and regularly collected in epidemiological studies. Such an index would be particularly valuable in instances when biomarker data are not readily available.

A 4- and 10-year mortality risk index has been developed for the general population of adults aged 50 years and older in the USA [5, 6]. It assigns a set number of points to a person based on his/her sociodemographic (age and sex), comorbid (diagnoses of cancer, diabetes, heart failure and lung disease), health behaviour (current smoking) and functional (activities of daily living) characteristics [5]. An individual's point score corresponds with his/her risk of 4- and 10-year mortality. However, this index is poorly calibrated to the older English population, where life expectancy is higher [7]. Middle-aged English adults tend to be healthier than American adults of the same age range, according to between-country differences in the prevalence of several chronic diseases and average levels of disease biomarkers [8]. While the biological processes underlying mortality risk are not expected to differ between the two countries, some predictive risk markers for mortality may differ based on between-country differences in health care systems, health behaviour distributions, early life exposures and health conditions, and the social determinants of health [8].

We aimed to develop and validate an index to predict 10-year mortality in the general population aged 50 years and older in England, using data from the English Longitudinal Study of Ageing (ELSA). This index is intended to be based on information readily ascertainable in a brief patient–physician discussion and to correspond to the existing American index, which was developed using data from the Health and Retirement Study (HRS), the sister study to the ELSA [9, 10].

## Methods

### Design

This study was conducted in accordance with the recommendations of the Transparent Reporting of a multivariable prediction model for Individual Prognosis Or Diagnosis (TRIPOD) statement [11]. Data were from 10,798 men and women aged 50 years and older in Wave 1 (2002–03) the population-based ELSA. The ELSA was established in 2002 based on a random stratified sample of households participating in the Health Survey for England [10]. In the first wave of ELSA, 11,391 core participants completed data collection (66% response rate). Of these, 10,798 consented to the mortality follow-up and were included in this study (94.8%). Data were collected biennially through home-based face-to-face interviews. The London Multicentre Research Ethics Committee granted ethical approval for the ELSA (MREC/01/2/91), and informed consent was obtained from all participants.

### Measures

The outcome of interest was all-cause mortality through March 2013. The National Health Service central data registry supplied mortality data for all participants who consented to the mortality follow-up. The mean follow-up period was 9.4 years (standard deviation: 2.6; range: 0–11 years). Consistent with the corresponding HRS index, we considered potential predictors of 10-year mortality in three categories: sociodemographic factors, health behaviour and comorbidity variables, and functional status variables. Detailed descriptions of these measures are available online in the Supplementary Material.

### Statistical analysis

We closely followed the statistical analysis plan used to develop mortality index by Lee *et al*. based on the HRS data [5]. The full detailed statistical analysis plan is available online in the Supplementary Methods. In brief, we randomly divided the 10,798 eligible and consenting participants into development (*n* = 5,377) and validation (*n* = 5,421) cohorts. In the development cohort, a Cox regression model was built to be robust to three selection strategies. Mortality risk point scores were assigned to the predictors in the final model by dividing the regression coefficient for each predictor by that of the predictor most weakly associated with all-cause mortality. Discrimination of the index was assessed using the area under the receiver operating characteristic (ROC) curves in the development and validation cohorts. Discrimination was also assessed across subgroups of age, ethnicity and education. A risk score–stratified Kaplan–Meier curve for all-cause mortality through March 2013 was calculated for the full cohort. The discriminatory capability of our model was compared to that of the original American HRS model using the chi-square test of equality of ROC areas. All statistical analyses were conducted using StataSE 13.1 (StataCorp, College Station, TX, USA).

## Results

Supplementary Table 1 shows the characteristics of the development and validation cohorts. Both cohorts were comparable on all characteristics. Overall, 10-year mortality was 24% (2,589/10,798). The unadjusted predictors of mortality risk in the development cohort are shown in Supplementary Table 2. All considered predictor variables were associated with mortality risk in unadjusted analyses, with the exception of body mass index (BMI). The final 10-item prediction model included the following variables: age, sex, vigorous physical activity, smoking, cancer, chronic lung disease, heart failure, difficulty preparing meals, difficulty pushing or pulling large objects and difficulty walking 100 yards (Tables 1 and 2). The fewest number of points achievable on the index is 0, corresponding to a woman aged 50–59 years who engages in some vigorous physical activity, does not smoke, has no diagnosis of cancer, chronic lung disease, or heart failure, and who has no difficulty preparing meals, pushing or pulling large objects or walking 100 yards. The maximum number of points achievable is 28.

**Table 1.** Independent risk factors for 10-year mortality in the development cohort and points allocated

Risk factors	Adjusted HR^a^ (95% CI)	Coefficients	Risk points^b^
Demographics			
Age			
60–64	1.72 (1.30–2.27)	0.560	1
65–69	3.33 (2.60–4.27)	1.310	3
70–74	6.15 (4.88–7.74)	2.029	5
75–79	10.29 (8.20–12.91)	2.767	7
80–84	17.37 (13.82–21.84)	3.583	9
≥85	26.93 (21.00–34.53)	4.492	12
Male	1.75 (1.56–1.96)	0.672	2
Health behaviours and comorbidities			
No vigorous physical activity	1.64 (1.41–1.91)	0.545	1
Smoking	1.89 (1.63–2.19)	0.813	2
Cancer	1.53 (1.26–1.85)	0.535	1
Chronic lung disease	1.41 (1.19–1.67)	0.608	2
Heart failure	2.45 (1.66–3.61)	1.426	4
Functional status variables			
Difficulty preparing meals	1.58 (1.28–1.94)	0.721	2
Difficulty pushing or pulling large objects	1.34 (1.16–1.55)	0.386	1
Difficulty walking 100 yards	1.44 (1.24–1.68)	0.534	1

HR, hazard ratio.

^a^All variables in column are mutually adjusted.

^b^Points calculated by dividing each beta coefficient by the lowest beta coefficient (difficulty pushing or pulling heavy objects) and rounding to the nearest integer value.

**Table 2.** Ten-year mortality index for older adults in England

1. Age _________________________	60–64:	1 point
	65–69:	3 points
	70–74:	5 points
	75–79:	7 points
	80–84:	9 points
	≥85:	12 points
2. Sex (male/female)	Male:	2 point
3. Do you take part in any sports or activities that are vigorous? (Y/N)	No vigorous physical activity:	1 point
4. Do you smoke at all nowadays? (Y/N)	Smoking:	2 points
5. Has a doctor ever told you that you have cancer or a malignant tumour (excluding minor skin cancers)? (Y/N)	Cancer:	1 point
6. Has a doctor ever told you that you have chronic lung disease, such as chronic bronchitis or emphysema? (Y/N)	Chronic lung disease:	2 point
7. Has a doctor ever told you that you have congestive heart failure? (Y/N)	Heart failure:	4 points
8. Because of a health or memory problem, do you have any difficulty preparing a hot meal? (Y/N)	Preparing meals:	2 points
9. Because of a health problem, do you have any difficulty pushing or pulling large objects like a living room chair? (Y/N)	Pushing/pulling large objects:	1 point
10. Because of a health problem, do you have any difficulty walking 100 yards? (Y/N)	Walking 100 yards:	1 point
	**Total points:**	_______

**Figure 1.**
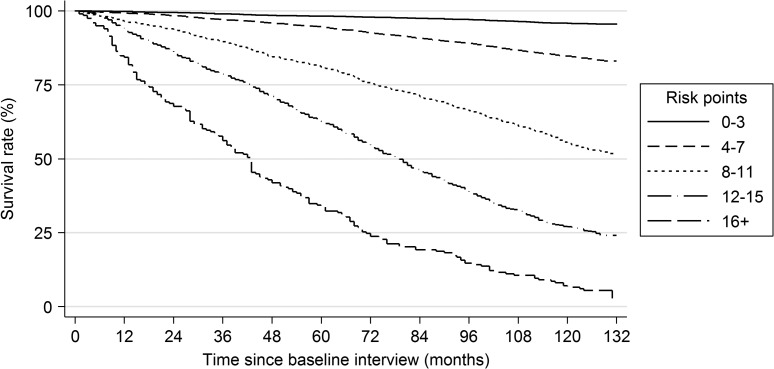
Kaplan–Meier survival curve by grouped risk points.

Mortality rates ranged from 1.9% (6/323; development cohort) and 1.5% (5/341; validation cohort) at the 0 point level to 96.9% (95/98; development cohort) and 93.1% (94/101; validation cohort) at the ≥16 point level (Supplementary Table 3). Few participants scored above 16 on the index and mortality rates were similar at and above this level; hence scores were collapsed at this point to maintain statistical power for the categorical analysis. The index showed excellent discrimination, with ROC area = 0.855 in the development cohort and ROC area = 0.859 in the validation cohort. Across specific population subgroups, the index showed good discrimination in the full cohort according to area under the ROC curve: 0.728 for ages 50–69 years, 0.679 for ages 70–79 years and 0.678 for ages ≥80 years; 0.902 for white adults and 0.846 for non-white adults; 0.840 for those with no educational qualifications, 0.859 for those with intermediate-level education and 0.846 for those with a higher degree. Figure 1 shows the Kaplan–Meier curve for survival through March 2013 in the entire cohort by grouped risk points. Although the HRS index showed no gross lack of fit in the ELSA sample (*P* = 0.166; Supplementary Table 4), it displayed significantly worse sensitivity and specificity for mortality than the new index (test of equality of ROC areas: *χ*^2^ = 60.62, *P* < 0.001; Supplementary Figure 1).

## Discussion

This simple 10-item index shows excellent discrimination and predictive capability for risk of 10-year mortality among English adults aged 50 years and older. The items included in the index are often collected in epidemiological studies of older adults and should be easily ascertainable during a brief patient–physician discussion. With slightly different risk point items and weightings, this index shows slightly improved sensitivity and specificity for 10-year mortality in the English population over a similar index developed for the older American population. This new mortality risk index is similar to frailty indices that predict mortality such as the Frailty Phenotype or the Frailty Index [12–14]; yet, it includes additional mortality-specific risk factors (namely age, sex and smoking status), is validated for use in the older English population and harmonised with an existing mortality risk index for the older American population. This index may be useful for research, policy and clinical purposes in England.

This new ELSA index and the original HRS index assigned the same number of risk points to variables for male, current smoking and lung disease. Both indices included diagnoses of cancer (1 point ELSA, 2 points HRS) and heart failure (4 points ELSA, 2 points HRS), difficulty pushing and pulling large objects (1 point ELSA, 2 points HRS) and difficulty walking 100 yards/several blocks (1 point ELSA, 2 points HRS). The ELSA index assigns a greater weighting to older age, with scores going up to 12 points for being aged 85 years and older versus 7 points for being aged 85 years and older in the HRS index. The ELSA index additionally includes no vigorous physical activity (1 point) and having difficulty preparing a hot meal (2 points). The HRS index additionally includes diabetes (1 point), BMI < 25 (1 point) and having difficulty bathing or managing finances (2 points each) [5]. The corresponding index items of age, sex, smoking status, heart failure, cancer, lung disease and difficulty walking make sense; the biological processes underpinning mortality risk are not expected to differ between the USA and England. Rather, differences in medical systems between the two countries may be an ecological contributor to some of the between-country differences we observed in predictors of mortality [15].

In the USA, Medicare is available at a monthly premium to American citizens aged 65 years and older who have paid Medicare taxes through work for at least 10 years [16]. In England, health care through the NHS is free at the point of care to adults of all ages [17]. In the USA, lack of health insurance coverage is consistently associated with increased risk of all-cause mortality and mortality due to diseases amenable to health care among working age adults [18–21]. Lack of adequate health insurance among some HRS cohort members, particularly in the pre-Medicare age group, may contribute to some observed differences in mortality and its predictors between the ELSA and HRS cohorts [19]. Uninsured patients with long-term chronic conditions have worse health outcomes in the USA than those with insurance [21]. Although the accumulated effect of inadequate health insurance on late life mortality is less well studied than mortality at working ages, it is plausible that a lifetime lack of adequate health care access would affect outcomes in later life, regardless of Medicare availability. The Affordable Care Act was introduced in 2010; its potential long-term benefits to population health have not yet come to fruition. Other factors that may explain differences in the two indices could be differential rates of some chronic conditions, such as diabetes [5, 22], and differential effects of various social determinants of health between the two countries [8].

Differences in the two indices with respect to BMI are more difficult to explain. BMI data in our cohort were missing at a higher rate than for other variables (33% missing; 3575/10798). We may have observed a lack of association between BMI and mortality for this reason although BMI is inversely associated with mortality only in adults older than 65 years [23, 24]. The ELSA cohort had a smaller proportion of adults older than 65 years than the HRS cohort, so this relatively weak predictor of mortality may have been more relevant in the HRS cohort. Mortality rates were lower among those with complete BMI data than in the full sample for this study, indicating that our data may not reflect the full distribution of BMI in the general population. This is a limitation of our study, as our index might have shown even better performance if BMI data were included. Regardless, the index shows high predictive capability for mortality without an objective BMI measurement, which might increase its ease of use in settings when BMI data are unavailable.

Our index is consistent with a recent index to predict 5-year mortality among adults aged 40–70 years in the UK Biobank [25]. Using data on 498,103 UK Biobank participants, Ganna and Inglesson developed 5-year mortality risk indices with 11 items for men and 13 items for women. They assessed 655 measurements for potential associations with mortality, while we aimed to develop and validate an index for an older segment of the population based on an *a priori* selected set of predictors. Similar methods were used in both studies, with concordant risk index items of age, diagnoses of cancer and heart conditions, and smoking [25]. The Biobank index includes regular pace of walking, while ours includes difficulty walking; their index additionally includes self-rated health and diagnosis of diabetes, along with car ownership and number of household members in men, number of live births in women and receipt of state benefits in men and women [25]. Each index has specific advantages. The Biobank Index is validated to predict 5-year risk of mortality among English adults aged 40–70 years, while the present ELSA index is validated to predict 10-year risk of mortality among adults aged 50 years and older, including those older than 70 years. Both indices are likely to be useful across research, policy and clinical settings for their respective purposes.

Our index has limitations. All variables were assessed through self-report in an in-person study interview and may be subject to recall error or bias. However, patient reports of chronic disease diagnoses and functional limitations have shown adequate validity in other similar samples [26, 27]. With the exception of BMI and history of falls, which was only measured among those aged 60 years and older, less than 2% of data were missing for all variables. There are likely to be other age-related risk factors for mortality not included in our model, as indicated by the large risk scores attributable to older ages, although a more complex model would reduce its practicality for applied use. Namely, ageing-related cognitive decline is a mortality risk factor that was excluded to avoid imposing the need for a cognitive assessment to be conducted for the index to be operational. The index may be statistically over-powered with respect to individual prognostication; so follow-up validation for individual prognoses within other prospective studies is needed. Follow-up for observed mortality with the HRS index has shown its predictions are valid in the American setting [28]. We did not have data on specific causes of death, which could be examined in relation to this index in the future. Finally, although older adults who were in permanent institutional care were excluded, the ELSA cohort is representative of the older population of England aged 50 years and older, including cognitively or physically impaired adults and those in hospital or temporary care [10].

In conclusion, we have developed and validated a simple yet comprehensive index to predict 10-year mortality risk among adults aged 50 years and older in England. Given that several ageing cohort studies exist in England (e.g. the ELSA, the Whitehall II cohort, the 1946 British Birth Cohort and the UK Biobank, as its sample ages), this index should be useful for investigators using the English data sets to study health during ageing, although it requires validation for individual prognostication in studies outside of the ELSA. The index includes mortality risk factors that should be easily discernible in a brief patient–physician discussion and are often available in epidemiological studies. The simplicity of the index is advantageous for accurate prediction of mortality risk when biomarker data are not available, and it should prove useful for several purposes.

Key points

- Ten-year mortality risk can be accurately predicted by 10 simple risk factors in older English adults.
- In this sample, 95% of adults with all 10 risk factors die within 10 years.
- This index should be useful for epidemiological research and clinical decision-making for older adults.

## Supplementary Material

Supplementary Data
