# Supplementary material for: The development and validation of an index to predict 10-year mortality risk in a longitudinal cohort of older English adults
Source: Age Ageing. 2016 Oct 28;46(3):427–32. doi: 10.1093/ageing/afw199 (PMC5405757; doi:10.1093/ageing/afw199)
Supplement: Supplementary Data [file afw199_SUPPLEMENTARY_DATA.docx]

SUPPLEMENTARY DATA

| Supplementary Table 1. Characteristics of study participants, English Longitudinal Study of Ageing, n=10,798 | | | |
| --- | --- | --- | --- |
| Characteristics | N (%) | | |
|  | Development cohort  (n=5377) | Validation cohort  (n=5421) | |
| **Demographics** |  |  | |
| Age |  |  | |
| 50-59 | 1980 (36.8) | 1983 (36.6) | |
| 60-64 | 820 (15.3) | 782 (14.4) | |
| 65-69 | 782 (14.5) | 855 (15.8) | |
| 70-74 | 674 (12.5) | 719 (13.3) | |
| 75-79 | 516 (9.6) | 516 (9.5) | |
| 80-84 | 387 (7.2) | 363 (6.7) | |
| ≥85 | 218 (4.1) | 203 (3.7) | |
| Women | 2934 (54.6) | 2945 (54.3) | |
| Education |  |  | |
| Higher degree | 1178 (21.9) | 1211 (22.4) | |
| Intermediate | 1879 (35.0) | 1942 (35.8) | |
| No qualifications | 2316 (43.1) | 2265 (41.8) | |
| Marital status |  |  | |
| Married | 3572 (66.4) | 3643 (67.2) | |
| Never married | 302 (5.6) | 278 (5.1) | |
| Divorced | 565 (10.5) | 573 (10.6) | |
| Widowed | 937 (17.4) | 926 (17.1) | |
| **Health behaviours and comorbidities** |  |  | |
| Smoking status |  |  | |
| Never or former smoker | 4375 (82.2) | 4419 (82.6) | |
| Current smoker | 949 (17.8) | 933 (17.4) | |
| Alcohol use |  |  | |
| Less than daily | 3828 (71.9) | 3846 (71.9) | |
| Daily or more | 1496 (28.1) | 1505 (28.1) | |
| Vigorous physical activity |  |  | |
| Some | 1967 (36.9) | 1963 (36.7) | |
| None | 3357 (63.1) | 3389 (63.3) | |
| Hypertension | 2042 (38.0) | 2052 (37.9) | |
| Diabetes | 400 (7.4) | 392 (7.23) | |
| Cancer | 331 (6.2) | 362 (6.7) | |
| Chronic lung disease | 375 (7.0) | 335 (6.2) | |
| Heart failure | 38 (0.7) | 41 (0.8) | |
| Other heart problems | 1089 (20.3) | 1185 (21.9) | |
| Stroke | 235 (4.4) | 246 (4.5) | |
| Psychiatric disease | 372 (6.9) | 396 (7.3) | |
| Memory-related disease | 39 (0.7) | 47 (0.9) | |
| Arthritis | 1827 (34.0) | 1713 (31.6) | |
| History of falls | 1047 (32.1) | 1021 (30.9) | |
| History of pain | 2075 (39.0) | 2049 (38.3) | |
| Incontinence | 838 (15.8) | 819 (15.3) | |
| Visual impairment | 208 (3.9) | 234 (4.3) | |
| Hearing impairment | 268 (5.0) | 294 (5.4) | |
| BMI <25^*^ | 1013 (28.3) | 1142 (31.4) | |
| **Functional status variables** |  |  | |
| Activities of daily living |  |  | |
| Bathing | 661 (12.3) | 664 (12.3) | |
| Dressing | 712 (13.2) | 723 (13.3) | |
| Toileting | 191 (3.6) | 197 (3.6) | |
| Eating | 83 (1.5) | 113 (2.1) | |
| Getting in or out of bed | 364 (6.8) | 351 (6.5) | |
| Walking across the room | 161 (3.0) | 196 (3.6) | |
| Instrumental activities of daily living |  |  | |
| Shopping | 490 (9.1) | 528 (9.7) | |
| Preparing meals | 220 (4.1) | 263 (4.9) | |
| Using the telephone | 112 (2.1) | | 88 (1.6) |
| Managing medications | 75 (1.4) | | 87 (1.6) |
| Managing finances | 124 (2.3) | | 136 (2.5) |
| Doing work around the house or garden | 850 (15.8) | | 883 (16.3) |
| Other functional variables |  | |  |
| Getting up from a chair after long periods | 1438 (26.7) | | 1382 (25.5) |
| Sitting for about two hours | 787 (14.6) | | 756 (14.0) |
| Walking 100 yards | 665 (12.4) | | 683 (12.6) |
| Pushing or pulling heavy objects | 959 (17.8) | | 992 (18.3) |
| Climbing 1 flight of stairs without resting | 803 (14.9) | | 815 (15.0) |
| Climbing several flights without resting | 1998 (37.2) | | 1927 (35.6) |
| Stooping, kneeling, or crouching | 1960 (36.5) | | 1902 (35.1) |
| Picking up a 5p coin from a table | 297 (5.5) | | 267 (4.9) |
| Reaching above one’s shoulders | 600 (11.2) | | 601 (11.1) |
| Lifting or carrying weights over 10lb | 1405 (26.1) | | 1384 (25.5) |
| Using a map | 275 (5.1) | | 304 (5.6) |

Note: Numbers may not sum to the total in each of the development and validation cohorts due to missing data for some variables.

^*^BMI = body mass index, calculated as weight in kilograms divide by the square of height in metres

| Supplementary Table 2. Bivariate analysis of risk factors for 10-year mortality in the development cohort | | |
| --- | --- | --- |
| Risk factors | Number (%) of deaths | Unadjusted HR (95% CI) |
| **Demographics** |  |  |
| Age |  |  |
| 50-59 | 117 (5.9) | 1.0 |
| 60-64 | 89 (10.9) | 1.91 (1.45-2.51) |
| 65-69 | 144 (18.4) | 3.35 (2.62-4.28) |
| 70-74 | 214 (31.8) | 6.29 (5.01-7.88) |
| 75-79 | 251 (48.6) | 10.94 (8.78-13.64) |
| 80-84 | 259 (66.9) | 17.70 (14.21-22.05) |
| ≥85 | 187 (85.8) | 32.04 (25.37-40.47) |
| Sex |  |  |
| Women | 632 (21.5) | 1.0 |
| Men | 629 (25.8) | 1.24 (1.11-1.38) |
| Education |  |  |
| Higher degree | 164 (13.9) | 1.0 |
| Intermediate | 350 (18.6) | 1.39 (1.15-1.67) |
| No qualifications | 747 (32.3) | 2.61 (2.20-3.09) |
| Marital status |  |  |
| Married | 645 (18.1) | 1.0 |
| Never married | 91 (30.1) | 1.77 (1.42-2.21) |
| Divorced | 100 (17.7) | 0.99 (0.80-1.22) |
| Widowed | 425 (45.4) | 3.04 (2.69-3.43) |
| **Health behaviours and comorbidities** |  |  |
| Smoking status |  |  |
| Never or former smoker | 992 (22.7) | 1.0 |
| Current smoker | 245 (25.8) | 1.16 (1.01-1.33) |
| Alcohol use |  |  |
| Less than daily | 917 (24.0) | 1.0 |
| Daily | 319 (21.3) | 0.88 (0.77-1.00) |
| Vigorous physical activity |  |  |
| Some | 226 (11.5) | 1.0 |
| None | 1011 (30.1) | 2.96 (2.56-3.42) |
| Hypertension |  |  |
| Absent | 693 (20.8) | 1.0 |
| Present | 568 (27.8) | 1.38 (1.24-1.55) |
| Diabetes |  |  |
| Absent | 1125 (22.6) | 1.0 |
| Present | 136 (34.0) | 1.64 (1.37-1.96) |
| Cancer |  |  |
| Absent | 1137 (22.5) | 1.0 |
| Present | 124 (37.5) | 1.93 (1.60-2.32) |
| Chronic lung disease |  |  |
| Absent | 1099 (22.0) | 1.0 |
| Present | 162 (43.2) | 2.32 (1.96-2.73) |
| Heart failure |  |  |
| Absent | 1234 (23.1) | 1.0 |
| Present | 27 (71.1) | 4.95 (3.38-7.25) |
| Other heart problems |  |  |
| Absent | 855 (19.9) | 1.0 |
| Present | 406 (37.3) | 2.12 (1.88-2.38) |
| Stroke |  |  |
| Absent | 1135 (22.1) | 1.0 |
| Present | 126 (53.6) | 3.09 (2.57-3.72) |
| Psychiatric disease |  |  |
| Absent | 1191 (23.8) | 1.0 |
| Present | 70 (18.8) | 0.76 (0.60-0.97) |
| Memory-related disease |  |  |
| Absent | 1237 (23.2) | 1.0 |
| Present | 24 (61.5) | 3.73 (2.49-5.59) |
| Arthritis |  |  |
| Absent | 733 (20.7) | 1.0 |
| Present | 528 (28.9) | 1.46 (1.31-1.63) |
| History of falls |  |  |
| Absent | 664 (30.0) | 1.0 |
| Present | 446 (42.6) | 1.56 (1.38-1.76) |
| History of pain |  |  |
| Absent | 694 (21.4) | 1.0 |
| Present | 543 (26.2) | 1.27 (1.13-1.42) |
| Incontinence |  |  |
| Absent | 983 (22.0) | 1.0 |
| Present | 251 (30.0) | 1.45 (1.26-1.66) |
| Visual impairment |  |  |
| Absent | 1145 (22.2) | 1.0 |
| Present | 116 (55.8) | 3.37 (2.79-4.08) |
| Hearing impairment |  |  |
| Absent | 1153 (22.6) | 1.0 |
| Present | 108 (40.3) | 2.08 (1.71-2.54) |
| BMI^*^ |  |  |
| ≥25 | 400 (15.6) | 1.0 |
| <25 | 150 (14.8) | 0.95 (0.79-1.15) |
| **Functional status variables** |  |  |
| Activities of daily living |  |  |
| Bathing |  |  |
| No difficulty | 934 (19.8) | 1.0 |
| Difficulty | 327 (49.5) | 3.15 (2.77-3.57) |
| Dressing |  |  |
| No difficulty | 976 (20.9) | 1.0 |
| Difficulty | 285 (40.0) | 2.17 (1.90-2.48) |
| Toileting |  |  |
| No difficulty | 1172 (22.6) | 1.0 |
| Difficulty | 89 (46.6) | 2.50 (2.02-3.11) |
| Eating |  |  |
| No difficulty | 1215 (23.0) | 1.0 |
| Difficulty | 46 (55.4) | 3.02 (2.25-4.06) |
| Getting in or out of bed |  |  |
| No difficulty | 1103 (22.0) | 1.0 |
| Difficulty | 158 (43.4) | 2.31 (1.96-2.74) |
| Walking across the room |  |  |
| No difficulty | 1166 (22.4) | 1.0 |
| Difficulty | 95 (59.0) | 3.65 (2.96-4.50) |
| Instrumental activities of daily living |  |  |
| Shopping |  |  |
| No difficulty | 1001 (20.5) | 1.0 |
| Difficulty | 260 (53.1) | 3.42 (2.98-3.92) |
| Preparing meals |  |  |
| No difficulty | 1131 (21.9) | 1.0 |
| Difficulty | 130 (59.1) | 3.74 (3.12-4.49) |
| Using the telephone |  |  |
| No difficulty | 1188 (22.6) | 1.0 |
| Difficulty | 73 (65.2) | 4.16 (3.28-5.27) |
| Managing medications |  |  |
| No difficulty | 1220 (23.0) | 1.0 |
| Difficulty | 41 (54.7) | 2.97 (2.18-4.06) |
| Managing finances |  |  |
| No difficulty | 1186 (22.6) | 1.0 |
| Difficulty | 75 (60.5) | 3.71 (2.94-4.68) |
| Doing work around the house or garden |  |  |
| No difficulty | 863 (19.1) | 1.0 |
| Difficulty | 398 (46.8) | 3.05 (2.71-3.44) |
| Other functional variables |  |  |
| Getting up from a chair after long periods |  |  |
| No difficulty | 770 (19.6) | 1.0 |
| Difficulty | 491 (34.1) | 1.92 (1.72-2.15) |
| Sitting for about two hours |  |  |
| No difficulty | 1040 (22.7) | 1.0 |
| Difficulty | 221 (28.1) | 1.28 (1.11-1.48) |
| Walking 100 yards |  |  |
| No difficulty | 920 (19.5) | 1.0 |
| Difficulty | 341 (51.3) | 3.34 (2.95-3.79) |
| Pushing or pulling large objects |  |  |
| No difficulty | 840 (19.0) | 1.0 |
| Difficulty | 421 (43.9) | 2.78 (2.47-3.12) |
| Climbing 1 flight of stairs without resting |  |  |
| No difficulty | 873 (19.1) | 1.0 |
| Difficulty | 388 (48.3) | 3.19 (2.83-3.60) |
| Climbing several flights without resting |  |  |
| No difficulty | 560 (16.6) | 1.0 |
| Difficulty | 701 (35.1) | 2.39 (2.14-2.67) |
| Stooping, kneeling, or crouching |  |  |
| No difficulty | 614 (18.0) | 1.0 |
| Difficulty | 647 (33.0) | 2.02 (1.81-2.26) |
| Picking up a 5p coin from a table |  |  |
| No difficulty | 1141 (22.5) | 1.0 |
| Difficulty | 120 (40.4) | 2.02 (1.67-2.44) |
| Reaching above one’s shoulders |  |  |
| No difficulty | 1054 (22.1) | 1.0 |
| Difficulty | 207 (34.5) | 1.72 (1.48-1.99) |
| Lifting or carrying weights over 10lb |  |  |
| No difficulty | 710 (17.9) | 1.0 |
| Difficulty | 551 (39.2) | 2.55 (2.28-2.85) |
| Using a map |  |  |
| No difficulty | 1131 (22.2) | 1.0 |
| Difficulty | 130 (47.3) | 2.66 (2.22-3.19) |

| Supplementary Table 3. Validation of the 10-year mortality risk index: comparing the model performance by point score in the development and validation cohorts and HRs for mortality risk at each point score | | | |
| --- | --- | --- | --- |
| Score | Development cohort  (n=5323)  No. died / No. at risk (%) | Validation cohort (n=5351)  No. died / No. at risk (%) | Whole sample (n=10674)  HR (95% CI) |
|  |  |  |  |
| 0 | 6/323 (1.9) | 5/341 (1.5) | 1.00 |
| 1 | 13/457 (2.8) | 12/478 (2.5) | 1.63 (0.80-3.30) |
| 2 | 28/656 (4.3) | 21/613 (3.4) | 2.36 (1.23-4.53) |
| 3 | 52/714 (7.3) | 51/694 (7.4) | 4.50 (2.42-8.38) |
| 4 | 45/473 (9.5) | 45/471 (9.6) | 5.97 (3.19-11.17) |
| 5 | 51/436 (11.7) | 65/461 (14.1) | 8.25 (4.44-15.31) |
| 6 | 79/406 (19.5) | 99/433 (22.9) | 14.15 (7.69-26.01) |
| 7 | 71/316 (22.5) | 91/310 (29.4) | 17.87 (9.70-32.91) |
| 8 | 137/357 (38.4) | 129/364 (35.4) | 27.30 (14.93-49.89) |
| 9 | 80/205 (39.0) | 111/246 (45.1) | 32.60 (17.75-59.87) |
| 10 | 153/300 (51) | 160/299 (53.5) | 44.09 (24.16-80.44) |
| 11 | 108/156 (69.2) | 102/142 (71.8) | 68.20 (37.18-125.1) |
| 12 | 108/156 (69.2) | 110/166 (66.3) | 66.32 (36.18-121.6) |
| 13 | 68/93 (73.1) | 62/73 (84.9) | 90.95 (49.13-168.4) |
| 14 | 90/117 (76.9) | 74/95 (77.9) | 87.84 (47.70-161.8) |
| 15 | 53/60 (88.33) | 55/64 (85.9) | 132.1 (71.03-245.8) |
| ≥16 | 95/98 (96.9) | 94/101 (93.1) | 184.9 (100.6-339.9) |
| ROC area | 0.855 | 0.859 | - |

| Supplementary Table 4. Cox regression model predicting 10-year mortality in the whole ELSA sample – based on the original HRS index | |
| --- | --- |
| Risk factors | Hazard ratio  (95% CI) |
| **Demographics** |  |
| Age |  |
| 60-64 | 2.01 (1.55-2.61) |
| 65-69 | 3.36 (2.65-4.26) |
| 70-74 | 6.78 (5.44-8.45) |
| 75-79 | 10.22 (8.18-12.78) |
| 80-84 | 20.17 (16.03-25.39) |
| ≥85 | 36.77 (28.12-48.09) |
| Male sex | 1.80 (1.60-2.03) |
| **Health behaviours and comorbidities** |  |
| Diabetes | 1.44 (1.21-1.73) |
| Cancer | 1.84 (1.50-2.26) |
| Chronic lung disease | 1.44 (1.19-1.74) |
| Heart failure | 1.85 (1.19-2.90) |
| BMI <25 | 1.10 (0.96-1.25) |
| Current smoker | 1.97 (1.69-2.29) |
| **Functional status variables** |  |
| Difficulty bathing | 1.30 (1.10-1.55) |
| Difficulty managing finances | 1.52 (1.09-2.10) |
| Difficulty walking 100 yards (several blocks) | 1.28 (1.07-1.54) |
| Difficulty pushing/pulling heavy objects | 1.29 (1.09-1.52) |


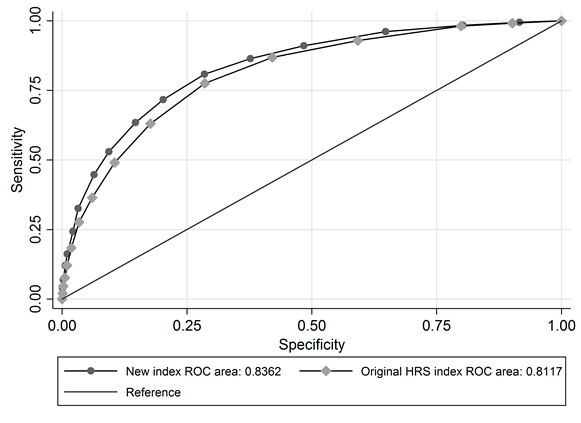


Supplementary Figure 1. Test of equality in ROC curve areas between the new ELSA and the original HRS index (χ² = 60.62, *P* < .001)
